# Pilot study investigating estrus length and estrus behavior in Norwegian Red cattle on a commercial dairy farm

**DOI:** 10.3389/fvets.2023.1219001

**Published:** 2023-09-12

**Authors:** Maien Munthe-Kaas, Guro Sveberg, Ingrid Hunter Holmøy, Elisabeth Kommisrud, Caroline Sorknes Haadem, Adam Dunstan Martin

**Affiliations:** ^1^Department of Production Animal Clinical Sciences, Norwegian University of Life Sciences, Ås, Norway; ^2^GENO Breeding and AI Association, Hamar, Norway; ^3^Department of Biotechnology, Inland Norway University of Applied Sciences, Hamar, Norway

**Keywords:** Norwegian Red, estrus behavior, estrus duration, estrus intensity, dairy cow

## Abstract

**Introduction:**

Norwegian Red has been shown to have high levels of estrus behavior under experimental conditions. However, the estrus behaviors of Norwegian Red cows have not been studied under commercial conditions.

**Methods:**

A herd of 89 Norwegian Red cows housed in free stalls on concrete, slatted floors, were continuously video monitored for 21 days. Ovarian cyclicity was confirmed in a final study sample group (*n* = 18) using milk progesterone concentrations. All mounting and standing activities were recorded, and the duration of mount estrus, standing estrus and the differences between these; prestand and poststand, were determined. The cycle stages metestrus, diestrus and proestrus were estimated based on the starting time and ending time of mount estrus.

**Results:**

All cows in the final study sample group exhibited the primary estrus sign, ‘standing to be mounted’ during estrus. Two (11%), eleven (61%) and six (33%) cows exhibited the behavior ‘standing to be mounted’ during metestrus, diestrus and proestrus, respectively. The number of mounts initiated by individual cows was higher during mount and stand estrus than during the rest of the estrous cycle. This study reports a median duration of mount estrus and stand estrus of 21.0 h (interquartile range (IQR) 15.0 to 27.3) and 14.3 h (IQR 12.0 to 18.8), respectively. The median counts per hour of all mount behaviors were 8.6 (IQR 5.6 to 11.3), 1.51 (IQR 0.3 to 3.8) and 1.7 (IQR 0.8 to 6.0) for standing estrus, prestand and poststand, respectively.

**Discussion:**

This study shows that under commercial conditions the Norwegian Red cow displays a high level of mount and stand activity associated with estrus.

## Introduction

Good reproductive performance is required to maintain the economic and environmental sustainability of dairy farming. Central to this is accurate estrus detection to facilitate the correct timing of artificial insemination. The estrus detection rate affects the calving-to-conception interval (1) and the calving interval (2). Accurate detection of estrus is also important to avoid inseminations during the luteal phase, abortions due to insemination during pregnancy (3) and, of course, to avoid missed insemination opportunities. Since around 1980, there has been a decline in dairy cow fertility internationally, especially in the Holstein-Friesian (HF) breed (4–6). For instance, conception rates in the United States dropped from approximately 55% in the 1950s, to around 45% for inseminations performed on spontaneous estrus, and 35% for timed AI, in the 1990s (6). In the United Kingdom, pregnancy to first service (%) dropped from 55.6 to 39.7% between 1975–1982 and 1995–1998 (4). However, recently the decline has stopped in many countries, after fertility traits were included in HF breeding programs (7). In Norway, the dominant dairy cow is the Norwegian Red (NR), which has had fertility included in its breeding program since 1972 (8), and the reproductive performance of NR cows compares favorably to HF cows (9, 10).

Standing to be mounted (STBM) is traditionally considered to be the primary sign of estrus, whereas the act of mounting another cow, or being mounted without a standing response, are some of numerous secondary estrous signs (11). High milk yields in lactating dairy cows have been associated with a lower estrus intensity, a lower total standing to be mounted time, and a shorter estrus duration (12). Estrus has been reported in HF cows to often occur without the expression of standing estrus, meaning that other behaviors might be more useful for estrus detection in this breed (13, 14). Other authors have suggested a stronger emphasis on the mount estrus period than on standing estrus for the HF breed (15). A point scoring system to be used by the farmer has been developed by Van Eerdenburg et al. (13) to facilitate estrus detection using secondary signs. In addition, there are several different technological aids for improving heat detection. These include tail-painting, pressure-activated heat mount detectors, pedometers, and radio telemetric devices (16), and are largely based on the detection of standing and mounting activity, as well as increased activity in general. With the emergence of precision farming (17) and implementation of different technologies on commercial farms internationally, knowledge of cattle behavior (both in estrus and not in estrus) in different breeds under common housing conditions is needed.

The duration of estrus and the intensity of estrous behaviors are known to differ between different breeds (18). In a study based on continuous surveillance, Sveberg et al. found that the NR cows both spend more time in sexually active groups, and mount and express STBM more often than HF. The duration of estrus was also longer for NR than for HF cows (15). However, Sveberg’s study was performed outdoors at a research farm in Ireland and does not reflect the conditions under which dairy cows are commonly kept in Norway, where the majority are housed indoors for most of the year. Several environmental and housing factors affect estrus expression in dairy cattle (19), and the frequency of STBM during observation sessions of 20 min have been shown to be decreased in cubicle housed HF cows compared to HF cows kept on pasture (20). Currently, there are no studies of NR behavior on commercial farms, despite the increasing footprint of the breed worldwide.

The objectives of this study were to determine the duration of estrus and to describe mounting and standing behavior throughout the estrous cycle for NR cows in a commercial Norwegian dairy herd.

## Materials and methods

### Animals

A commercial Norwegian dairy herd of 11 heifers and 78 cows was observed continuously for 21 days in October, during the housing season. The cow’s herd consisted of 78 cows in 1st, 2nd and over 2nd lactation, with a distribution of 43, 13, and 22 cows, respectively. A group of cows that could be expected to show estrus were selected for this study. Their milk was analyzed to measure progesterone concentration to confirm the occurrence of estrus. Cows selected for sampling were minimum 5 weeks postpartum. Exclusion criteria were reproductive disorders postpartum, insemination prior to the start of the study, pregnancy and moderate or severe lameness - a lameness score of over 2/5 in the week preceding the study’s start (21). To ensure that there would be cows in estrus during the study period, an estrus synchronization program consisting of two intra-muscular injections of 0.5 mg cloprostenol (Estrumate, MSD Animal Health) 11 days apart was commenced 21 days before the start of the study on cows that calved 7–10 weeks before study start. Progesterone sampling to confirm cyclicity was not performed after the treatment. The study group (*n* = 27) included 14, 2 and 11 cows in 1st, 2nd and over 2nd lactation, respectively.

### Housing and management

The cows were housed in a free-stall barn with a concrete slatted floor, and 84 cubicles. The cows were milked twice daily, at 06:00–07:20 and 17:00–18:20, in a rotary parlor with 16 milking stalls. Forage was fed twice daily and consisted of brewer’s grains and grass silage *ad libitum*. Concentrates were fed, according to yield, by automated concentrate feeders placed in the free-stall area.

### Progesterone sampling and analysis

Samples for the determination of progesterone concentration in milk were taken by the farmer from cows in the selected study group, during every milking. Whole milk from included cows was sampled using a milk meter, ensuring samples representative for the entire milking event, and a bronopol tablet was thereafter added to the 20 mL sample. The samples were labeled immediately and kept at 4°C prior to freezing at −18°C on the day of collection. The samples were transported to the Norwegian School of Veterinary Science for analysis. Using enzyme immunoassay (22) and the second-antibody coating technique (23), the progesterone concentrations in the samples were determined. The samples were analyzed in duplicate, and the intra-assay coefficient of variation for progesterone concentration in whole milk was less than 10%. At progesterone concentrations in whole milk of 1.5 and 19.7 ng/mL, the inter-assay coefficients of variation were 9.2 and 5.3%, respectively. Using a 20 μL sample, the limit of sensitivity was less than 10%.

### Behavior recording and data management

The 89 cows in the herd were marked individually prior to and during the study using white water paint on red and black hair coat and black paint on white hair coat every second or third day. The marking consisted of a number code unrelated to expected estrus and was the basis of the blinded study, in which the video observer had no knowledge of the estrous cycle stage of the individual study subjects. The behavior of all the cows in the herd was recorded. However, only events in which at least one cow from the selected study group (*n* = 18) was participating, is described in this study.

Five cameras (Axis 211, Axis Communications, Lund, Sweden) were placed to assure the whole area available to the cows was under surveillance. One picture was taken by each camera every second, stored as. jpg files, and saved on an internal hard drive in the camera. Photos were taken of every cow to be able to verify identification during video analysis. The program AXIS Camera Control (Axis Communications) was used to review the video files and record the activity of every cow according to the descriptors in section 2.5 (below) using a Microsoft Office Access Database (Microsoft Corp., Redmond, WA). The video was reviewed by two researchers individually, who then compared results. In the case of discrepancy both researchers looked at the video clip together and determined which behaviors were being displayed and which cows were involved. The final agreement represented the behavior and individual that was recorded in the final study dataset.

### Mount and stand behavior

All mount and stand behaviors involve one initiating (I) and one receiving (R) cow. The definitions of mount and stand behaviors were modifications based on the definitions used by Sveberg et al. (24), and were as follows:

1. Standing to be mounted (STBM): R makes no effort to escape when mounted by I. The mount must last for 2 s or longer to be defined as a stand event. A cow remaining stationary when mounted head end on or sideways, was also considered to be standing;
2. Mount with standing reaction (STBM seen from the perspective of the initiating cow): I mounts R from behind, head end on, or sideways, and R makes no attempt to escape. The mount must last for 2 s or longer to be defined as a stand event;
3. Restricted standing to be mounted (rSTBM): An event where R makes no attempt to escape when being mounted by I in the cubicle or at the eating front and the mounting lasts for 2 s or longer. The freedom of movement is considered as being restricted;
4. All STBM: STBM + rSTBM;
5. Mount: A mount without standing reaction; I successfully mounts R from behind, where both legs are resting on R’s rump;
6. Head mount: I mounts R head-end on;
7. Side mount: I mounts R sideways;
8. Mount attempt (unsuccessful): I tries to mount R by raising the front limbs, but fails;
9. All mounting events: Sum of all mounting and standing events; STBM, rSTBM, mount, head mount, side mount and mounting attempts.

### Stages of estrous cycle

Each cow was defined to be in the luteal phase if the progesterone concentration in milk was 3 ng/mL or higher in samples from two consecutive milkings, or 5 ng/mL or higher in one sample (4, 22, 25, 26). The definitions of the different stages of the estrous cycle were as follows:

1. Mount estrus (MTE): the interval from the first to the last mount engaged in by the cow in estrus, confirmed by progesterone concentrations <3 ng/mL during this period (24). A consecutive period of minimum 2 mounts within 12 h were required. Clusters of 2 or more mount/stand events which were of either a shorter duration than 12 h or an intensity of less than 1 event per 2 h, and separated from a longer and more intense consecutive period of mounting/standing events by more than 12 h were not included in the mount estrus;
2. Stand estrus (STE): the interval from the first to the last received standing event, in which the estrus cow makes no effort to escape when mounted by other cows. The interval may coincide with, or be shorter than, MTE (24);
3. Prestand: the interval between the beginning of MTE and the beginning of STE (27);
4. Poststand: the interval between the ending of STE and the ending of MTE (27);
5. Metestrus: the first 48 h after the ending of MTE (28);
6. Proestrus: the last 72 h before the start of MTE (28);
7. Diestrus: the interval between 48 h after the ending of MTE and 72 h before the start of MTE. If the study was completed during a cow’s luteal phase, data from the previous estrous cycle were added.

**Figure 1:**
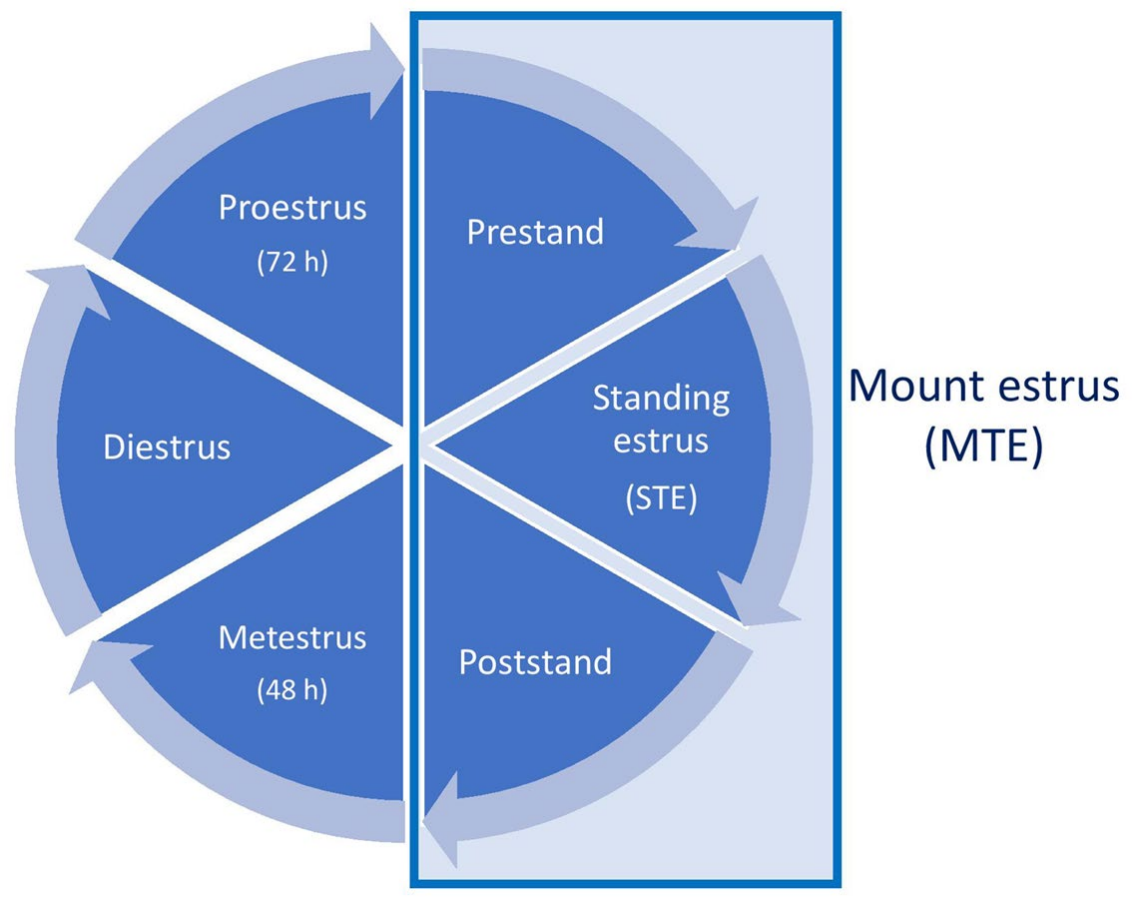
Illustration of the estrous cycle timeline (not to scale). The mount estrus (MTE) period includes prestand, standing estrus (STE), and poststand, and is followed by metestrus, diestrus, and proestrus. After one complete cycle, a new MTE begins.

An illustration of the timeline of the different phases of the estrous cycle is presented in Figure 1.

### Descriptive statistics

Continuous variables were assessed for normality visually using histograms. The results of non-normally distributed variables are expressed as median values and interquartile range (IQR). Normally distributed variables are expressed as mean values ± standard deviation (SD). Data management and analyses was performed using Stata version 16.0 (StataCorp, College Station, Texas 77,845, USA).

## Results

### Study group

Eighteen of the 27 selected cows were included in the study results. The reasons nine cows were excluded are as follows; one was slaughtered 7 days after the start of the study, two were excluded as purulent vaginal discharge was diagnosed in the study period, two cows’ samples were not analyzed due to missing information, three of the sampled cows were excluded because the study period started and/or ended in the middle of their estrus periods, and one cow was excluded due to sampling error.

The final study group (*n* = 18) included nine, two and seven cows in first, second and third or more lactation, respectively. The mean daily milk yield of the 18 cows included in the study was 24.4 ± 4.6 (±SD) kg milk. All the cows in the final study group were characterized as having cyclical ovarian activity based on milk progesterone concentration measurements, and all expressed MTE and STE.

### Duration of the phases of the estrous cycle

From the first measured progesterone concentration < 3 ng/mL until the start of MTE, there was a median of 32 h, with a range from 20 to 87 h (IQR 26–47). The time from the end of MTE to the last measured progesterone concentration < 3 ng/mL was a median of 61 h, with a range from 25 to 94 (IQR 58–68). The median values of the durations of the estrus phases; MTE, STE, prestand and poststand, are presented in Table 1.

**Table 1:** The median duration of estrus phases in hours (*n* = 18), range and interquartile range (IQR).

Estrus phase	Median (h)	Range (h)	IQR (h)
MTE	20.97	12.25–39.92	14.95–27.28
STE	14.25	3.44–28.40	11.99–18.80
Prestand	0.90	0.00–10.06	0.00–5.81
Poststand	2.89	0.00–23.19	0.00–8.52

MTE, mount estrus; STE, standing estrus; Prestand, the period in MTE before STE starts; Poststand, the period in MTE after STE ends.

### Mount and stand activity during estrus

**Figure 2:**
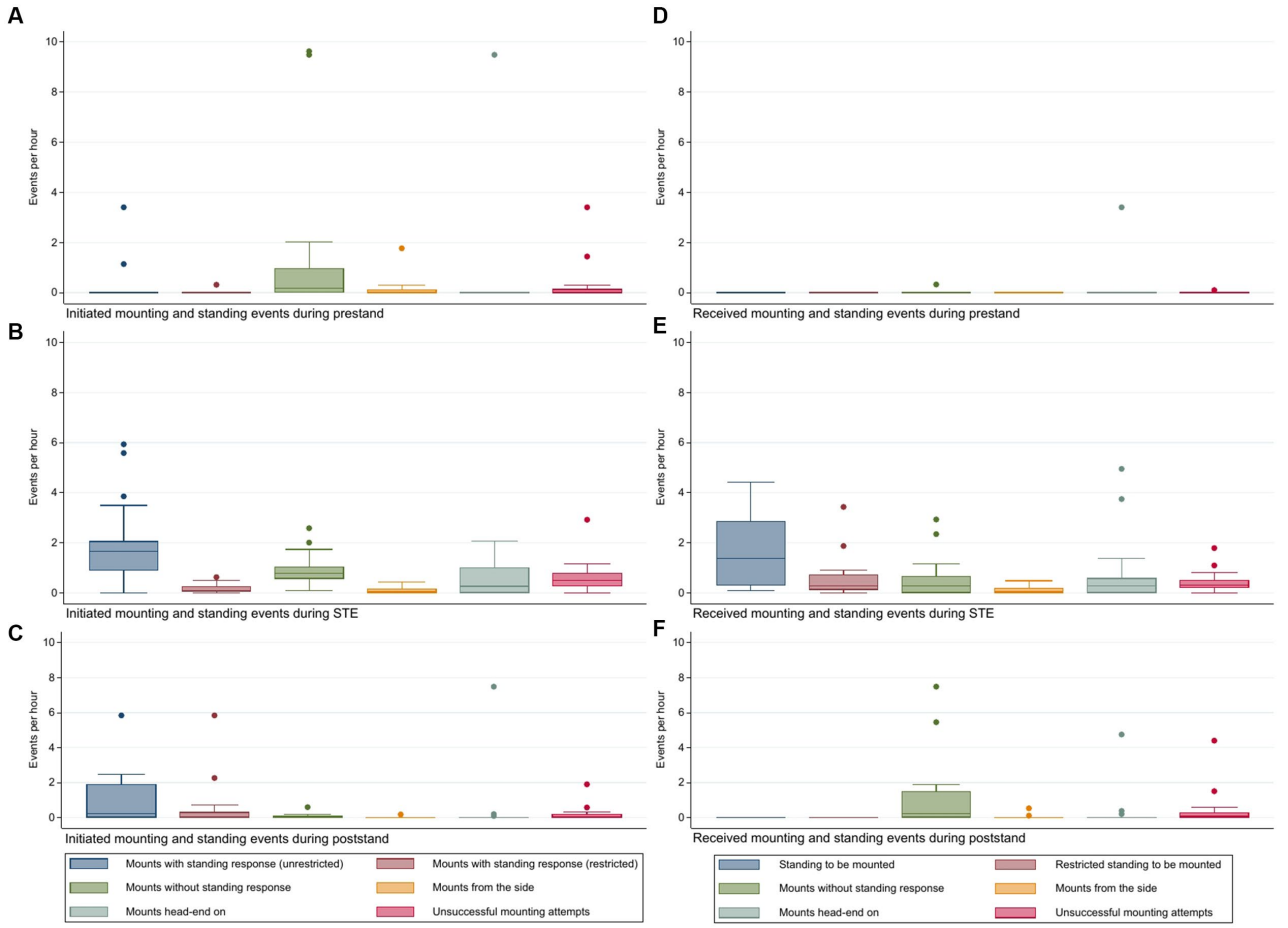
Count of initiated estrus behaviors per hour by **(A)** prestand, **(B)** standing estrus, and **(C)** poststand, and count of received estrus behaviors per hour by **(D)** prestand, **(E)** standing estrus, and **(F)** poststand.

All the cows in the final study group expressed both MTE and STE. STBM and ‘all STBM’ accounted for 34 and 49% of all the mounts received during MTE, respectively. Four cows expressed STBM, and one cow expressed rSTBM as their first mounting activity during MTE (*n* = 13 for prestand). Two and three cows expressed STBM and rSTBM as their last mount activity during MTE, respectively (*n* = 13 for poststand). The total number of times a cow participated in mount activities during the estrus phases and the count per hour (cph) during these phases are presented in Table 2. In Table 3, the total numbers of initiated and received mounting and standing behaviors during the estrus phases are presented. Figure 2 presents the cph of initiated and received mounts and stand behaviors during prestand, STE and poststand.

**Table 2:** Median total counts and counts per hour (cph), range and interquartile range (IQR) of all mount and stand events participated in during the different estrus phases, including events which resulted in a standing response and unsuccessful mounting attempts, both initiating and receiving behaviors.

	Median	Range	IQR
Total count of all mount events			
MTE (*n* = 18)^1^	159.5	10–401	74–180
STE (*n* = 18)^1^	140.5	9–293	69–171
Prestand (*n* = 18)^1^	1	0–15	0–3
Poststand (*n* = 18)^1^	5	0–107	0–8
Cph, all mount events			
MTE (*n* = 18)^1^	5.48	0.67–14.70	3.79–11.16
STE (*n* = 18)^1^	8.60	0.98–15.91	5.63–11.26
Prestand (*n* = 13)^1^	1.51	0.12–18.97	0.29–3.75
Poststand (*n* = 13)^1^	1.72	0.36–18.82	0.80–6.03

For the total count *n* = 18 for all phases. The calculation of cph is based on the actual number of individuals expressing each phase of estrus.

^1^ MTE, mount estrus; STE, standing estrus; Prestand, the period in MTE before STE starts; Poststand, the period in MTE after STE ends.

**Table 3:** Median total counts, range and interquartile range (IQR) of initiating and receiving mount and stand behaviors during the different estrous phases (*n* = 18).

		Initiating behaviors			Receiving behaviors		
		Median	Range	IQR	Median	Range	IQR
MTE^1^	All mount behaviors*	74	5–140	45–107	62.5	5–275	25–102
	All STBM**	31.5	0–95	15–53	27.5	5–106	10–59
	STBM^2^	23.5	0–85	14–51	16	2–88	4–44
	rSTBM^2^	2	0–13	1–5	4.5	0–73	2–15
	Mount^2^	13	2–38	10–17	6.5	0–85	1–10
	Sidemount^2^	1.5	0–7	0–4	1	0–12	0–3
	Headmount^2^	5	0–45	0–16	5	0–96	0–10
	Mounting attempts^2^	8.5	1–48	4–18	7	0–58	2–9
STE^1^	All mount behaviors	68	4–139	40–104	59.5	5–189	25–95
	All STBM	26.5	0–84	14–53	27.5	5–106	10–59
	STBM	23	0–78	13–51	16	2–88	4–44
	rSTBM	1.5	0–7	1–4	4.5	0–73	2–15
	Mount	10.5	1–33	7–15	6	0–54	0–9
	Sidemount	1	0–7	0–4	1	0–9	0–3
	Headmount	5	0–44	0–14	5	0–69	0–10
	Mounting attempts	8.5	0–48	4–13	4.5	0–33	2–7
Prestand^1^	All mount behaviors	1	0–13	0–2	0	0–2	0–0
	All STBM	0	0–7	0–0	0	0–0	0–0
	STBM	0	0–7	0–0	0	0–0	0–0
	rSTBM	0	0–1	0–0	0	0–0	0–0
	Mount	0.5	0–7	0–2	0	0–2	0–0
	Sidemount	0	0–1	0–0	0	0–0	0–0
	Headmount	0	0–2	0–0	0	0–1	0–0
	Mounting attempts	0	0–5	0–0	0	0–1	0–0
Poststand^1^	All mount behaviors	2	0–21	0–7	1	0–86	0–4
	All STBM	0.5	0–18	0–6	0	0–0	0–0
	STBM	0	0–14	0–2	0	0–0	0–0
	rSTBM	0	0–6	0–1	0	0–0	0–0
	Mount	0	0–3	0–1	0	0–31	0–2
	Sidemount	0	0–1	0–0	0	0–3	0–0
	Headmount	0	0–1	0–0	0	0–27	0–0
	Mounting attempts	0	0–2	0–1	0	0–25	0–2

^1^ MTE, mount estrus; STE, standing estrus; Prestand, the period in MTE before STE starts; Poststand, the period in MTE after STE ends.

^2^ STBM, Standing to be mounted; rSTBM, Standing to be mounted at eating front or in cubicle (restricted STBM); Mount, mounting from behind without standing response; Sidemount, mounting from the side without standing response; Headmount, mounting head-end on without standing response; Mounting attempts, one cow attempts to mount another cow, but does not succeed.

* including mounts without standing response, sidemounts, headmounts, mounting attempts and all mounts which resulted in a standing response.

** including STBM and rSTBM.

### Mount and stand activity during metestrus

During metestrus, nine of the 18 cows (50%) were mounted by another cow up to four times. These mounts resulted in a standing response once or twice for two of the 18 cows (11.1%). Six of the 18 cows (33.3%) in the study group mounted another cow once or twice during metestrus.

### Mount and stand activity during diestrus

#### Received mounts

During diestrus, 14 cows (77.8%) were mounted by another cow, between one and 103 times, whereof 13 cows were mounted between one and 24 times. These mounts resulted in a standing response for 11 of the cows (61.1%), which stood to be mounted between one and 21 times. Of these STBM events, 86.7% were unrestricted. Ten cows (55.6%) were mounted from behind without a standing response between one (*n* = 7) and 23 times during diestrus, and six cows (33.3%) received one unsuccessful mount attempt each. Two cows were mounted head-end on two and 44 times, and five cows (27.8%) were mounted sideways between one and seven times during diestrus.

#### Initiated mounts

Eleven cows (61.1%) mounted another cow during diestrus, between one and 97 times, of which 10 cows mounted another cow between one and 12 times. Mountings by the initiating cow resulted in a stand response from the receiving cow between one and 97 times for seven of the cows (38.9%). Of these seven, six mounted another cow between one and 12 times. During the same period, five cows (27.8%) mounted another cow without a standing response between one and seven times. Only one cow mounted another cow sideways once, two cows mounted another cow head-end on once and twice, and three cows had one or two unsuccessful mount attempts each during diestrus. The cow which was mounted 103 times during diestrus was the same cow as mounted another cow 97 times during the same period.

### Mount and stand activity during proestrus

During proestrus, nine cows (50%) were mounted by another cow one to eight times in total. These mounts resulted in a standing response for six of the cows (33.3%), which stood to be mounted one to seven times during the same period.Of these STB events, 76.5% were unrestricted. Seven cows (38.9%) mounted another cow once or twice during proestrus.

## Discussion

### Expression of the primary estrus sign, standing to be mounted

In contrast to studies in other breeds all cows in this study expressed the primary sign of estrus, standing to be mounted (STBM), when in estrus (13, 14, 29). The number of times each cow expressed STBM during STE in this study was higher than previously reported in continuous studies for other breeds (14, 15, 30), but similar to the report for NR outdoors in Ireland (15). The high number of cows expressing STBM in this study indicates that onset of standing activity could be a more useful predictor of ovulation time for NR cows compared to HF cows where its usefulness has been questioned (29). However, STBM was observed during all four stages of the estrous cycle (estrus, metestrus, diestrus, proestrus) in this study, with more than 60% of the cows expressing the primary estrus sign during diestrus.

Standing to be mounted is traditionally considered the most reliable sign of a cow being in estrus (13, 18, 31) and has previously been reported rarely to happen when cows are not in estrus (29, 32). Traditionally the occurrence of STBM has been regarded as evidence of a cow being in estrus (13, 14). However, in this study such an interpretation would have led to many false-positive results, meaning that factors such as intensity of estrous signs should be considered. Inseminations during the luteal phase and during pregnancy have previously been reported (3, 4), indicating the occurrence of behaviors typically associated with estrus in pregnant cows and cows in diestrus. The reason for the observed high incidence of these behaviors during diestrus in the current study is unclear. Estrus expression is highly dependent on the concentration of circulating estradiol and the relationship between estradiol and progesterone (31, 33). One possible explanation could be that the cows were providing moderate milk yield (mean daily milk yield 24.4 ± 4.6 (±SD) kg) and therefore may have had follicles which produced more estradiol than those in higher yielding cows, or because of a lower steroid metabolism in relatively low yielding dairy cows (34). Further studies, preferably with transrectal ultrasound monitoring and estrogen and progesterone sampling throughout the study period, would be useful to better understand this finding.

### Initiated mounts

Initiating mount behavior is considered to be an accurate sign of a cow being in or close to estrus (32), but has also been reported to occasionally occur during diestrus (13). In this study, cows expressed initiating mount behavior during all four phases of the estrous cycle, but with a considerably lower intensity during metestrus, diestrus and proestrus than during estrus. This suggests that the intensity of initiating mounts, in addition to expression of STBM, could be useful for correctly characterizing estrus in the NR cow. Esslemont and Bryant (35) have previously defined a cow as being in estrus if she mounted another cow six times or more in a 24 h period. More recently, in a study using continuous video monitoring to observe estrus behaviors in HF cows, the mean occurrence of initiated mounts was 6.6 (range 0–22) on the day of estrus (14). In this study, one cow initiated 97 mounts during diestrus. While the reasons for this are unknown it illustrates the potential importance of individual thresholds been set in precision farming estrus detection systems to optimize their sensitivity and specificity. Sensor technology has the possibility to measure every mount a cow makes. Therefore, it is important to investigate further the natural variation in mount behaviors throughout the estrous cycle under common conditions, to be able to adequately perform estrus detection with these devices.

### Duration of estrus

The present study supports previous evidence that NR cows have long MTE and STE periods (15). The duration of MTE in this study (21 h) is considerably longer than mean duration of behavioral estrus reported for HF cows in previous studies, of between 11 and 14 h (13, 14, 29). While the duration of STE (14 h) in this study population is similar to the durations reported for HF cows in the seventies, eighties and early nineties (13, 24, 29), but longer than the more recent results (36). Milk yields are negatively associated with the duration of standing estrus (9) and so the lower production of NR cattle could partially explain the reduction in the duration of estrus in HF compared to NR cows. However, in a study comparing HF and NR differences in estrus duration were present, despite milk yield and environmental conditions been similar (15).

The reason for this difference has not been investigated by this study. However, the similarity of the current NR’s STE to those of historic studies in HF is intriguing and requires further investigation. Our hypothesis is that including reproductive traits in the breeding program of the NR since the 1970s (37) combined with the low use of reproductive synchronization programs in Norway (38) has meant that the duration of estrus has been preserved, as it has been critical to breeding success in Norway. Further work is required to test this hypothesis.

### Intensity of estrus

The high cph found in this study suggests a high intensity of mount behaviors in NR during estrus, despite being housed on concrete floors, which have been negatively associated with such behaviors (6, 19). However, because hormonal estrus synchronization was performed prior to the study, it is reasonable to assume that there might have been a higher number of cows in estrus at the same time, than in a normal situation in a commercial farm. This could have skewed the results toward a higher intensity of estrus expression due to the formation of sexually active groups. Despite this, the intensity of estrus signs, measured as median counts per hour (cph) of mount events during MTE, in this study were similar to the means previously reported for NR (15). A long duration and intensity of estrus could make estrus easier to detect during traditional observing periods but is also important to consider when adapting estrus detection technologies for use in NR herds. However, cph is an average measure and therefore does not reflect any periods of higher or lower intensity during the defined period. It is therefore possible that periods of intense mounting and standing activity could be missed by farmers depending on visual estrus detection, particularly if they do not spend sufficient time.

### Applicability and limitations

The cows in this study were kept under typical conditions for dairy cows in Norway and were minimally disturbed during data collection, as estrus detection was based on video surveillance. A strength of this study is that the housing environment and management of the herd was typical for NR herds in Norway. This contrasts with the study previously performed comparing estrus in NR and HF cows in Ireland where the cows were kept outdoors in a restricted area and in an experimental setting (15). Further, cows across many parities were included in this study, although no heifers were included.

Undoubtedly the study was limited by the low number of animals remaining in the study group after exclusions were made. The small numbers prevented meaningful statistical analysis from being performed, making the study descriptive in nature. Further, only healthy cows were included in the study. A number of diseases are known to negatively influence display of estrus, e.g., lameness (39), so by selecting a healthy population the study does not accurately represent a commercial dairy herd, with inherent endemic disease. Unfortunately, the effect of number of cows in estrus on mount and stand activity and on the participation of diestrous cows in sexually active groups (40) could not be evaluated in the current study, due to the high number of cows with unknown cycle status in the herd. It is possible that by synchronizing the cows before the study start that an ‘artificial’ sexually active group was created. Further studies, including all cows in a herd, are needed to better understand these behaviors.

Despite these limitations the findings of the current study largely support the previous findings for NR by Sveberg et al. (15) and we believe the results of this study are applicable to commercial NR dairy cows.

## Conclusion

This study describes estrus duration and mount and stand activity throughout the estrous cycle for NR cows in a commercial Norwegian dairy herd kept under typical conditions. The current study provides new knowledge of the occurrence of mount and stand behaviors during other phases of the estrous cycle than estrus itself, and a high number of individuals expressed the primary estrus sign also during diestrus. This means that farmers should not base the decision to inseminate on one observed sign of estrus alone. The duration of estrus was longer than described for other dairy breeds, the cows initiated a high number of mounts when in estrus, and all included cows expressed the primary estrus sign, standing to be mounted.

## Data availability statement

The raw data supporting the conclusions of this article will be made available by the authors, without undue reservation.

## Ethics statement

Ethical approval was not required for the studies involving animals in accordance with the local legislation and institutional requirements because this was an observational study with minimal disturbance of the animals. Written informed consent was obtained from the owners for the participation of their animals in this study.

## Author contributions

MM-K, GS, EK, and AM contributed to conception of the study. EK conducted the project administration and funding acquisition. GS collected the data. MM-K and IH curated the data. MM-K, IH, and AM performed the formal analysis. MM-K and AM designed the methodology and wrote the original draft of the manuscript. CH and AM supervised the project. All authors critically reviewed the text, read, and agreed to the published version of the manuscript.

## Funding

This work was funded by Geno Breeding and AI Association, and the Research Council of Norway, grant no. 173974/I10 “Oestrus and Oestrus Behavior in NRF and Holstein Breeds in Modern Cattle Housing Systems.” It also forms part of the MM-K’s residency for the European College of Animal Reproduction.

## Conflict of interest

The authors declare that the research was conducted in the absence of any commercial or financial relationships that could be construed as a potential conflict of interest.

## Publisher’s note

All claims expressed in this article are solely those of the authors and do not necessarily represent those of their affiliated organizations, or those of the publisher, the editors and the reviewers. Any product that may be evaluated in this article, or claim that may be made by its manufacturer, is not guaranteed or endorsed by the publisher.
